# Hotel Performance in the UK: The Role of Information Entropy in a Novel Slack-Based Data Envelopment Analysis

**DOI:** 10.3390/e23020184

**Published:** 2021-01-31

**Authors:** Yong Tan, Ali Jamshidi, Abdollah Hadi-Vencheh, Peter Wanke

**Affiliations:** 1 Department of Accounting, Finance and Economics, Huddersfield Business School, University of Huddersfield, Huddersfield, Queensgate HD1 3DH, UK; 2 Department of Mathematics, Isfahan (Khorasgan) Branch, Islamic Azad University, Isfahan 19585/466, Iran; ali.jamshidi@khuisf.ac.ir (A.J.); ahadi@khuisf.ac.ir (A.H.-V.); 3 COPPEAD Graduate Business School, Federal University of Rio de Janeiro, Rio de Janeiro 21941-901, Brazil; peter@coppead.ufrj.br

**Keywords:** UK hotels, data envelopment analysis, information entropy, cash flow generation, operating scale, learning process

## Abstract

Previous hotel performance studies neglected the role of information entropy in feedback processes between input and output management. This paper focuses on this gap by exploring the relationship between hotel performance at the industry level and the capability of learning by doing and adopting best practices using a sample of 153 UK hotels over a 10-year period between 2008–2017. Besides, this research also fills a literature gap by addressing the issues of measuring hotel performance in light of negative outputs. In order to achieve this, we apply a novel Modified slack-based model for the efficiency analysis and Least Absolute Shrinkage and Selection Operator to examine the influence of entropy related variable on efficiency score. The Results indicate that less can be learnt from inputs than from outputs to improve efficiency levels and resource allocation is more balanced than cash flow and liquidity. The findings suggest that market dynamics explains the cash flow generation potential and liquidity. We find that market conditions are increasingly offering the opportunities for learning and improving hotel efficiency. The results report that the distinctive characteristic of superior performance in hotel operations is the capability to match the cash flow generation potential with market opportunities.

## 1. Introduction

The tourism and hotel management literature has long praised the benefits of hospitality and leisure issues on customer satisfaction levels [1] and the resulting impact on performance [2,3,4,5] but most research has been limitedly based on individual hotel performance. Hence, there is a lack of studies looking at industry performance that can reflect learning process and cash flow generation from operating scale that could not be measured or captured by analyzing each firm alone [6,7]. Despite this call for further investigation on hotel performance, and the recognition of learning processes as a key theme for improving cash flow generation [8], we identify a research gap concerning whether information entropy related-variables can lead to different performance results at the hotel level within the industry ambit.

Performance in hotel industry is often characterized by concentration of best practices in few large players with high economies of scale and profitability [9]. Big international hotel chains are particularly representative of this context. Conversely, local hotel chains suffer from small operational scale and working capital constraints, which limits their innovation rate and their ability to adequate to best practices over the course of time [10].

United Kingdom, as one of the European countries with a long history, special heritage and traditional culture, attracts huge amount of tourists every day. This tourist attraction was facilitated by the capital of UK, London, which is one of the most important financial centers in the world. In addition, as the increase in the economic growth and optimization in the production process derived from the advancement of internet technology and information sharing, people across the globe saw an increase in the amount of disposable income. Therefore, people do not only pursue the possession of physical capital, but focuses more towards the wealth of themselves at the spiritual level. In other words, people nowadays tend to spend money on exploring the interesting and different aspects in their life. One area of these would be travelling to different countries to feel different culture, food, people, et cetera. The popularity of travelling undoubtedly boosts the development and prosperity of the hotel industry, which is one of the most important components in the hospitality sector, and further contribute to the country’s economic growth.

The importance of the hotel industry in the economy together with the increase in the tourism volume induce more researchers to investigate the performance in the hotel industry. On top of the research studies using relevant accounting ratios to evaluate the profitability in the hotel sector, a growing number of research articles use advanced operational method to assess the efficiency level of hotels which provided more accurate results comparing to the accounting ratios. Besides the traditional non-parametric Data Envelopment Analysis and parametric Stochastic Frontier Analysis, scholars built upon and extend these methods resulting in a number of innovative models including Data Envelopment Analysis metafrontier, Data Envelopment Analysis window analysis, Bayesian Stochastic Frontier Analysis, triangular Data Envelopment Analysis, super efficiency Data Envelopment Analysis and grey entropy, slack-based Data Envelopment Analysis, network Data Envelopment Analysis, stochastic Data Envelopment Analysis, hyperbolic network Data Envelopment Analysis as well as two-layer bootstrapped Data Envelopment Analysis.

The objective and contribution of this study is to explore the relationship between hotel performance at the industry level and the capability of learning by doing and adopting best practices. Precisely the study is based on the underlying hypothesis that entropy-related variables could help in increasing cash flow generating potentials by a better use of current productive scale [11,12]. Besides, this research also fills a literature gap by addressing the issues of measuring hotel performance in light of negative outputs, which is typical of cash-flow and liquidity related variables. To this end, a novel Modified slack-based model is developed to allow better discrimination of super-efficiency scores. Finally, we propose the Least Absolute Shrinkage and Selection Operator to examine the influence of entropy related variable on efficiency scores. 

Hotel performance measurement is a key research area surrounded by epistemic uncertainty with respect to the dual formed not only by the geographic and temporal scope of the analysis, but also by the method chosen to measure performance. In a broader sense, epistemic uncertainty reflects the lack of prior knowledge on how hotel performance could be impacted by economic conditions (contextual variables) scattered through time and space. For many practical purposes, epistemic uncertainty is inherent to delimitating the object-method pair under study and manifests itself regardless of the literature gap identified, the scale used to measure variables, the variable (input/output) selection, and the reproducibility conditions that are intrinsic to some extent to the hoteling industry, which may vary within and between countries. For instance, there is no questioning that the proper identification of the literature gap is relevant for advancing the body of knowledge in hotel performance, especially with respect to DEA models where a plethora of different alternatives could be employed to handle specific aspects of hotel industry efficiency in different countries. However, research gaps and input/output measurement scales do not by themselves mitigate epistemic uncertainty in the ambit of hotel performance, but only assure under the aspects of internal validity—in light of the current body of knowledge and scale validity—that the proper analytical models were developed to adequately handle the specific nature of what is being measured.

Hence, as regards this paper, epistemic uncertainty can be conceptualized as the scientific uncertainty in the process of modelling hotel performance criteria in distinct geographic locations over the course of time. Despite limited data and knowledge, the joint use of information theory and soft computing offers methodological tools to mitigate epistemic uncertainty while increasing research validity and reproducibility: (i) sensitivity analysis not only by running alternative models, but also by allowing the incorporation of variables with negative values in DEA; and (ii) using information entropy principles for improving decision-making as regards whether or not a given model is contributing to reduce epistemic uncertainty by mapping how improvements in hotel inputs and outputs could be achieved through learning from other variables. The methodology here employed satisfy these two conditions, not only by increasing the valid scale range of inputs and outputs to allow negative values in DEA, but also by the composing the information entropy of efficiency scores, inputs, outputs, and contextual variables into its major constituents, thus offering to academics and practitioners an improvement roadmap in the hotel industry. 

The results show that information entropy on inputs is much wider than the one of outputs, therefore, less can be learnt from inputs to improve the efficiency level in the UK hotel industry. In addition, we find that the entropy dispersion of the contextual and business-related variables is in between the one of inputs and outputs, thus, something can be learnt from the market dynamics related to tourism inflows and outflows as well as financial conditions of hotels, which is related to the cash flow generation potential. We further notice that the evolvement of inputs and contextual variables related to information entropy goes in opposite directions, in other words, at the time when information entropy for inputs increases, the one for contextual variables decreases, which indicates and conforms that over the examined period, there is a degree of opportunity to learn from market conditions to improve the hotel efficiency level. Finally, the negative impact of input/output joint entropy on super efficiency scores suggest that the UK hotels should be operated under a decreasing return to scale combined with a price discount policy, which would be helpful to improve efficiency level from the perspective of increase the cash flow generation potential. 

The remainder of this paper is organized in four sections. First, we provide a discussion of the literature concerning estimation of hotel efficiency. Then, we describe the study’s methodology. Next, we offer the results and discussion. Finally, we conclude with implications, limitations and future research opportunities.

## 2. Literature Review 

The efficiency in the hotel industry has been extensively investigated by the empirical literature. The original operational methods used to estimate hotel efficiency can mainly divided by Data Envelopment Analysis [13,14,15,16,17,18,19,20,21] and Stochastic Frontier Analysis [22]. However, Data Envelopment Analysis suffers from a number of limitations [23,24,25]: (1) the effect of exogenous variables on the operation is ignored; (2) statistical errors are ignored; (3) statistical test with the results are difficult to be performed; (4) it cannot clearly indicate the way to improve efficiency. In comparison, the main weakness of Stochastic Frontier Analysis is it needs a particular parametric function form to represent the underlying technology and distributional assumption for the efficiency terms are required [26]. 

Recently, a number of research articles extended the traditional Data Envelopment Analysis or Stochastic Frontier Analysis by introducing some advanced techniques in estimating the level of efficiency in the hotel industry. This includes the use of (1) Data Envelopment Analysis metafrontier analysis [27], this method benefits from the advantage of being able to compare the performance between different groups without any ignorance of heterogeneity between them [28]; however, the existing study suffers from the drawbacks of being unable to integrate the meta-frontier and undesirable output together; (2) Data Envelopment Analysis window analysis [29,30]; this method has the advantage of making it feasible to evaluate and compare the performance of Decision Making Units in different periods through regarding them as a separate entity in different periods [31], however, Data Envelopment Analysis window analysis suffers from the limitations that this technique was designed for a short period of time and the random error in the variables was not considered and the dependence structure to estimate the efficiencies was not used [32].

Besides the above, the empirical studies also proposed and present the following methods including (3) Bayesian stochastic frontier analysis [33,34], in the Bayesian approach, a special function form for the production frontier and a source of randomness is needed [35] although this method has the benefit of being able to separate the efficiency from the technological difference and include the prior information about the parameter in inference [36]; (4) Triangular Data Envelopment Analysis model [37], this specific method has the advantage of considering efficiency and effectiveness in the hotel production process at the same time under a model [38], however, as argued by the authors themselves, due to the fact that Data Envelopment Analysis requires large number of observations, in particular when the number of inputs and outputs increase, therefore, Data Envelopment Analysis is not suitable for small hotel chains. 

In addition, we also observe that empirical studies have attempted to use the method below including (5) super efficiency Data Envelopment Analysis and grey entropy [39], as argued by the authors, the super-efficiency Data Envelopment Analysis gives the efficient unit a score of more than 1 and less efficient ones with a score of less than 1 and it is a better method in efficiency measurement in practice and grey entropy benefits from the advantage that being able to compute the weight without any rigorous statistical requirement and assumptions; however, super-efficiency Data Envelopment Analysis suffers from the issue of infeasibility [40]; (6) Slacked-based Data Envelopment Analysis model [41]; this method benefits from the advantages of providing more discriminatory power and more sources of inefficiency can be detected [42]; however, the study failed to identify and consider the internal sub-production process (i.e., divide the production process into several stages).

Third, a number of research articles have applied the following method in estimating hotel efficiency including (7) Network Data Envelopment Analysis model [43,44]; the network Data Envelopment Analysis supplements the previous slacks-based model by dividing the production into stages and being able to identify the source of (in)efficiencies for each of the stages [45], however, under the assumption of variable returns to scale, the network DEA does not provide information on divisional efficiency but only provide information on projection frontier [46]; (8) Stochastic Data Envelopment Analysis model [47,48]; as argued by [47], the stochastic Data Envelopment Analysis model improves the conventional Data Envelopment Analysis model by providing a higher ability to measure efficiency in the environment with uncertainty. On the other hand, the drawback of this method lies to the fact that stochastic variables are imposed limitations (i.e., the stochastic inputs are normally distributed) [49]. 

Finally, we also find that the following methods have been applied in the hotel performance evaluating including (9) Hyperbolic network Data Envelopment Analysis model [50]; as argued by the authors, this method benefits from the advantages of being able to not only allow the inefficient Decision Making Units to projects their inputs and outputs to the efficient frontier simultaneously, but consider the shared inputs in the model as well. However, a non-linear programming problem has to be solved for using the hyperbolic efficiency measure [51]; (10) two-layer bootstrapped Data Envelopment Analysis model [52]. As argued by the authors, this method uses an independent and repeated sampling process, through which the errors could be reduced. [53] argue that bootstrapped Data Envelopment Analysis model has unreasonable weights assigned for inputs and outputs which would affect the robustness of the results. 

Regarding the inputs and outputs selection in the hotel production process, quite a few different inputs have been proposed and used by various studies including accommodation capacity [54]; Tourist arrivals [55]; number of employees [30,56]; Labor cost [57,58]; depreciation expenses [16]; capital [13,59]; operating expenses [60]; among others, on the other hand, there are quite a few outputs considered by the empirical research such as revenue [61,62]; average occupancy rate [63,64]; average room rate [65]; rate of satisfaction [16]; Sales [66]; among others.

In summary, through reviewing the literature regarding the estimation of efficiency in the hotel industry, we observed that there has been no attempt to examine the relationship between hotel performance at the industry level and capability of learning by doing and adopting best practices, the investigating of this is of particular importance regarding the potential behavior that can be engaged in by the hotels through interacting with other hotels in the industry. We fill in this gap by examining this relationship. The issue of negative output in the efficiency analysis has not been addressed in the estimating of hotel efficiency, we contribute to the empirical literature by proposing a modified slack-based model. Finally, we contribute to the literature in the examination of hotel efficiency by being the first to propose a Least Absolute Shrinkage and Selection Operator to examine the influence of entropy related variable on efficiency scores which is supposed to provide more robustness and accurate results for policy making purposes. 

## 3. Methodology 

This section describes the major methodological cornerstones employed in this research. Readers should recall from the introduction that the hybrid approach (DEA-Information Entropy-LASSO regression) here discussed is designed towards the mitigation of epistemic uncertainty in hotel performance. Being a non-parametric technique, DEA embedds in itself a large degree of uncertainty, as long as no distributional assumptions are required whatsoever with respect to inputs, outputs, and the resulting computed scores. Information entropy allows a distributional assessment of these quantities, helping in understanding not only their degree of reliability, but also in unveinlyng non-linear associations that may exist among them, moving further beyond the traditional correlation coefficient approach. In turn, LASSO allows the identification of the critical explanatory variables, by imposing informational penalties, helping in structuring by means of a linear model how much of impact on efficiency scores is due to a given information entropy subset of variables, i.e., inputs, outputs, and contextual variables.

### 3.1. The Data

We collect a dataset of 153 UK hotels over a 10-year period between 2008–2017. Regarding the selection of inputs and outputs for the novel modified slack-based model proposed, we choose three inputs to represent the operational scale which are number of employees, depreciation/amortization, as well as cost of goods sold, we consider five outputs to represent the cash flow generation potential including operational revenue, other operating expenses, liquidity ratio, capitalization as well as hotel size. Capitalization is measured by the ratio between shareholders’ funds and total assets, hotel size is measured by the natural logarithm of total assets. The third group of variables besides inputs and outputs are contextual business-related variables such as number of international arrivals, number of international departures, international tourism expenditures, international tourism receipts, total assets as well as shareholders’ fund. 

Cost of goods sold, operating revenue, depreciation/amortization, operating expenses, total assets are in the unit of thousand Euros, hotel size, capitalization and liquidity ratios are in ratios, international tourism receipts and expenditure are in the unit of current US dollars, number of arrivals and number of departures are in the unit of persons. The input and output variables together with total assets, shareholders’ fund are collected from Amadeus database (https://www.bvdinfo.com/en-gb/our-products/data/international/amadeus), which contains comprehensive information about large public and private companies across Europe, while other contextual and business related variables including tourism arrival, departure, expenditure and receipts in the UK are collected from the world bank database (https://data.worldbank.org/). 

We use cost of goods sold, depreciation/amortization and the number of employees to generate five outputs including operating revenue, other operating expenses, liquidity ratio, capitalization and hotel size. The selection of cost of goods sold is in line with [17], while the cost of goods sold includes the cost of labor. We include the number of employees as another input, in line with [13], who used labor cost and employees number at the same time. We argue that the labor input in the hotel efficiency analysis should focus on two different perspectives, namely the labor cost in monetary terms and the number of employees. Finally, we consider the depreciation/amortization as one input is in accordance with the opinion of [67] who argues that the inputs can be generally divided into assets-related inputs and cost-related inputs, the former one contributes to the costs indirectly through depreciation and amortization. 

With regard to the selection of output variables, normally RevPAR, which is a traditional measure in hotel performance should be included, however, the Amadeus database through which we collected our dataset did not provide the statistics for this variable, therefore, we used the operating revenue as an alternative indicator. Basically, these operating revenue and RevPAR are similar from the perspective that both of them measure the revenue from the accommodation services provided by the hotels. We used other operating expenses as one of the outputs, this is in contrast with the previous literature which treated it as an input variable [39], we are the pioneer to consider it as an output due to the consideration that hotels aim to minimize the overhead in hotel operation, this is also related to one of our contributions which is to measure hotel performance in light of negative outputs. Another aim of hotel operation is to achieve growth, therefore, we use hotel size as one of the output variables. We consider the capitalization as one of the output variables considering the fact that hotel operation aims to achieve a higher profitability as well as improve the level of stability, a higher level of capitalization serves the purpose of reducing hotel risk. Finally, our consideration to use liquidity ratio as one of the output variables is in line with [44]. 

We control for a number of contextual variables including the number of international arrivals, number of international departures, international tourism receipts and international tourism expenditure. The number of international arrivals is an important factor that will contribute to performance improvement in the hotel industry [68]. In terms of the outbound international tourism because some flights will be in the morning time, this will make hotel accommodation a necessity for the domestic tourists, therefore, we argue that the international tourism departure will have a potential impact on hotel performance. International tourism expenditure is supposed to influence the hotel’s cash flow generation potential because nearly half of tourism expenditure is related to accommodation and lodging [69]. Ref. [70] uses tourism receipt as the output variable and two input variables (namely, the number of tourist bed-nights in hotels and the number of tourist bed-nights in campsites) to measure tourism productivity. This shows that there is a linkage between hotel accommodation and tourism receipts. In other words, tourism receipts will boost the hotel’s cash flow potential. We also include hotel size as one of the contextual variables following [71] considering the fact that large hotels would be able to reduce the cost from economies of scale. Finally, we include the shareholders fund as the final contextual variable. Large shareholders’ fund reduces the borrowing cost and further facilitate the improvement in hotel performance. 

### 3.2. The Novel Modified Slack-Based Model

#### 3.2.1. Preliminaries

Suppose we have a set of n Decision Making Units, ${\mathrm{Decision}\;\mathrm{Making}\;\mathrm{Unit}}_{j}$, j=1,2,…,n, Such that each one uses m inputs $\left\{x_{ij}\right\}$, i=1,2,…,m to produce s outputs $\left\{y_{rj}\right\}$, r=1,2,…,s. We show ${\mathrm{Decision}\;\mathrm{Making}\;\mathrm{Unit}}_{j}$ by $\left(x_{j},y_{j}\right)$, j=1,2,…,n, and let $X=\left(x_{ij}\right)\in {\mathbb{R}}^{m\times n}$ and $Y=\left(y_{rj}\right)\in {\mathbb{R}}^{s\times n}$ be the input and output matrix, respectively. Besides, we assume that:

(a) There is at least one positive output.
(b) There is at least one positive input.
(c) There is some negative input/output data.

The production possibility set is defined as below

$$P=\left\{\left(x,y\right)\;|\;x\geq X\lambda \;,\;y\leq Y\lambda \;,\;\overset{n}{\underset{j=1}{\sum }}\lambda_{j}=1\;,\;\lambda \geq 0\right\}$$

where $\lambda \in {\mathbb{R}}^{n}$ is the intensity vector.

The Modified Slack-based Model is defined as follow [72]:

$$\begin{array}{ll} \min & \rho =\frac{1-\sum_{i=1}^{m}\frac{w_{i}s_{i}^{-}}{P_{io}^{-}}}{1+\sum_{r=1}^{s}\frac{v_{r}s_{r}^{+}}{P_{ro}^{+}}}\\ & s.t.\;\sum_{j=1}^{n}\;\lambda_{j}x_{ij}+s_{i}^{-}=x_{io}\;\;\;\;i=1,2,\ldots ,m \end{array}$$

(1) $$\overset{n}{\underset{j=1}{\sum }}\;\lambda_{j}y_{rj}+s_{r}^{+}=y_{ro}\;\;\;\;r=1,2,\ldots ,s$$

$$\begin{array}{l} \sum_{j=1}^{n}\;\lambda_{j}=1\\ \sum_{i=1}^{m}\;w_{i}=1\\ \sum_{r=1}^{s}\;v_{r}=1\\ s_{i}^{-},w_{i}\geq 0\;\;\;i=1,2,\ldots ,m\\ s_{r}^{+},v_{r}\geq 0\;\;\;r=1,2,\ldots ,s\\ \lambda_{j}\geq 0\;\;\;\;\;\;\;j=1,2,\ldots ,n \end{array}$$

where $P_{io}^{-}=x_{io}-\underset{j}{\min}\left\{x_{ij}\right\},\;i=1,2,\ldots ,m$ and $P_{ro}^{+}=y_{ro}-\underset{j}{\max}\left\{y_{rj}\right\},\;r=1,2,\ldots ,s$.

#### 3.2.2. The Proposed Model

In this section we propose our ranking approach. First, we compute the efficiency score of Decision Making Units using the Modified Slack-Based Model. If there is just one efficient Decision Making Unit, then we do not need ranking. On the other hand, suppose that there is more than one efficient unit and let ${\mathrm{Decision}\;\mathrm{Making}\;\mathrm{Unit}}_{o}$ be one of the efficient units. Besides, let $X_{o}$ and $Y_{o}$ be the inputs/outputs matrices excluding ${\mathrm{Decision}\;\mathrm{Making}\;\mathrm{Unit}}_{o}$, respectively. Hence, the new production possibility set is

$$P^{o}=\left\{\left(x,y\right)\;|\;x\geq \overset{n}{\underset{j=1,j\neq o}{\sum }}\lambda_{j}x_{j},\;y\leq \overset{n}{\underset{j=1,j\neq o}{\sum }}\lambda_{j}y_{j}\;,\;\overset{n}{\underset{j=1,j\neq o}{\sum }}\lambda_{j}=1\;,\;\lambda_{j}\geq 0\;,\;\forall jo\right\}$$

Now, the proposed super-efficiency model is as follows

$$\begin{array}{ll} \min & \rho_{o}=\frac{1+\sum_{i=1}^{m}\frac{w_{i}t_{i}^{+}}{P_{io}^{-}}}{1-\sum_{r=1}^{s}\frac{v_{r}t_{r}^{-}}{P_{ro}^{+}}}\\ & s.t.\;\sum_{j=1,j\neq o}^{n}\lambda_{j}x_{j}\leq x_{io}+t_{i}^{+}\;\;\;i=1,2,\ldots ,m \end{array}$$

(2) $$\overset{n}{\underset{j=1,j\neq o}{\sum }}\lambda_{j}y_{j}\geq y_{ro}-t_{r}^{-}\;\;\;r=1,2,\ldots ,s$$

$$\begin{array}{l} \sum_{j=1,j\neq o}^{n}\lambda_{j}=1\\ t_{i}^{+}\geq 0\;\;\;i=1,2,\ldots ,m\\ t_{r}^{-}\geq 0\;\;\;r=1,2,\ldots ,s\\ \lambda_{j}\geq 0\;\forall j\neq o, \end{array}$$

here $\left(t_{i}^{+},\;t_{r}^{-}\;\right)$ show the deviation of $\left(x_{io},\;y_{ro}\right)$ from the frontier of the production possibility set such that ${\mathrm{Decision}\;\mathrm{Making}\;\mathrm{Unit}}_{o}$ situated outside of the production possibility set. It is clear that if $\rho_{o}^{*}=1$ then $t_{r}^{-*}=0$ and $t_{i}^{+*}=0$ and this means that ${\mathrm{Decision}\;\mathrm{Making}\;\mathrm{Unit}}_{o}$ is a non-extreme efficient Decision Making Unit. When $\rho_{o}^{*}>1$ then at least one component of $t^{-*}$ ($t^{+*}$) is nonzero and in this case ${\mathrm{Decision}\;\mathrm{Making}\;\mathrm{Unit}}_{o}$ is an extreme efficient unit.

**Remark** **1.** *If* $\underset{j}{max}\left\{y_{rj}\right\}=y_{ro}\neq 0$ *or* $\underset{j}{min}\left\{x_{ij}\right\}=x_{io}\neq 0$ *then we set* $P_{ro}^{+}=\left|y_{ro}\right|$ *or* $P_{io}^{-}=\left|x_{io}\right|$ *respectively, and as a result,* $P_{ro}^{+}$ *and* $P_{io}^{-}$ *are positive.*

The model (2) has the following properties.

**Proposition** **1.** *Let*$\rho_{o}^{*}$be the optimal value of model (2). Then,$\rho_{o}^{*}\geq 1$.

**Proof.**  The proof is clear and hence omitted. □

**Proposition** **2.** *The super-efficiency score* $\rho_{o}^{*}$ *is units invariant, i.e., it is independent of the units in which the inputs and outputs are measured provided these units are the same for every Decision Making Unit.*

**Proof.**  The proof is clear and hence omitted. □

**Proposition** **3.** *Model (2) is translation invariant.*

**Proof.**  The proof is clear and hence omitted. □

Model (2) is a fractional programming and can be easily converted to the following linear model:

$$\begin{array}{ll} \min & \delta_{o}=k+\sum_{i=1}^{m}\frac{w_{i}T_{i}^{+}}{P_{io}^{-}}\\ & s.t.\;k-\sum_{r=1}^{s}\frac{v_{r}T_{r}^{-}}{P_{ro}^{+}}=1\\ & \;\sum_{j=1,j\neq o}^{n}\mu_{j}x_{j}\leq x_{io}+T_{i}^{+}\;\;\;i=1,2,\ldots ,m \end{array}$$

(3) $$\overset{n}{\underset{j=1,j\neq o}{\sum }}\mu_{j}y_{j}\geq y_{ro}-T_{r}^{-}\;\;\;r=1,2,\ldots ,s$$

$$\begin{array}{l} \sum_{j=1,j\neq o}^{n}\mu_{j}=1\\ T_{i}^{+}\geq 0\;\;\;i=1,2,\ldots ,m\\ T_{r}^{-}\geq 0\;\;\;r=1,2,\ldots ,s\\ \mu_{j}\geq 0\;\forall j\neq o,\\ k\geq 0 \end{array}$$

where $=\frac{1}{1-\sum_{r=1}^{s}\frac{v_{r}t_{r}^{-}}{P_{ro}^{+}}}$, $T_{i}^{+}=kt_{i}^{+}$, $T_{r}^{-}=kt_{r}^{-}$, $\mu_{j}=k\lambda_{j}$ for each i, r and j.

**Example** **1.** *This example has been taken from [72]. Consider Table 1. In this table we have 13 Decision Making Units each one uses two inputs $\left(x_{1},x_{2}\right)$ to produce three outputs $\;\left(y_{1},y_{2},y_{3}\right)$. One input ($x_{1}$) and one output ($y_{1}$) is positive and other data are negative. Table 2 displays the obtained results for this example. In this table, we computed the efficiency score of each Decision Making Unit using model (1). This table contains the super-efficiency score and the rank of each unit using the proposed model (model (3)), as well. It is clear from the Table 3 that C, G, H, K and M are efficient Decision Making Units by model (1) and this model could not rank these efficient Decision Making Units. On the other hand, the new model not only computes the efficiency scores but ranks also the efficient Decision Making Units. Besides, Sharp’s model cannot be applied to computing multi-period efficiency.*

### 3.3. Novel Modified Slack-Based Data Envelopment Analysis Applied to UK Hotel Efficiency

Information Entropy can be conceptualized as a measure of uncertainty, which is a probabilistic concept. Depending on the entropy characteristics, the randomness and dispersion produced by a random variable can be determined by calculating the information entropy for each original variable—for example: input, output—under each group of analysis (hotel, city, and year). The greater the value of the information entropy, the greater the randomness or the dispersion within the ambit of each group and, therefore, the greater the heterogeneity produced in analyzing this unknown phenomenon [73]. In this paper, information entropy is used to analyze the aggregate input, output, and contextual variable distributions based on data collected at the hotel level, for city, for year. Through this approach, a novel assessment is established in estimating the inherent heterogeneity of a given locus of analysis with respect to each hotel epistemic uncertainty in translating operational scale into cash flows.

Joint Entropy, in turn, represents the joint epistemic uncertainty of two random variables. It helps in understanding how balanced their responses are. Specifically, with respect to input and output Joint Entropy at the hotel level, it is possible to describe whether cash flow generating potentials are elastic or inelastic to variations in operational scale. This is particularly important because hotel operations are capital intensive requiring somewhat higher breaking-even levels. Joint Entropy between outputs and contextual variables related to tourism inflow may reflect also whether or not revenues are elastic with respect to demand. Differently from Information Entropy and Mutual Information, which is discussed next, Joint Entropy bridges the gap between epistemic uncertainty and learning processes by imposing economic constraints or boundaries—such as scale and revenue elasticities—on possible joint fluctuations. While balanced joint fluctuations would denote a neutral or constant response; unbalanced ones would represent decreasing/increasing returns-to-scale and inelastic/elastic revenue responses to demand given a capacity level.

Mutual Information represents the mutual information level of two random variables A and B and measures the “amount of information” that can be inferred about one random variable by observing the other [74]. Putting it into other words, mutual information is intricately linked to the expected “amount of information“ held in a random variable, which is not necessarily limited to a linear dependence like the correlation coefficient or other forms of unidirectional causality [75] That is why mutual information is also known as information gain [76]. This means that mutual information bridges the gap between elements in distinct sets (in this research, input, output, and contextual variable sets). The information gain for one random variable from learning for the other could even be as high enough to offset their individual entropies. This would be equivalent to affirm that mutual information represents the strength or intensity of the feedback processes that exists between the inputs and outputs observed at each Decision Making Unit level, by which information is also gained on how improving cash flow generation through learning on how managing operational scale (and vice-versa).

**Figure 1:**
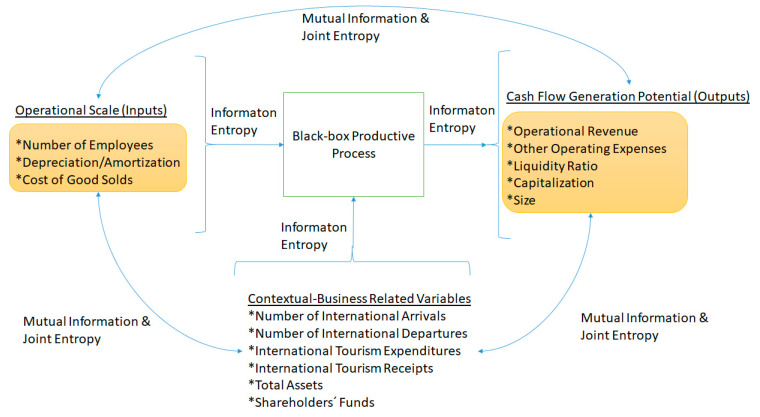
Role of information entropy on UK hotels efficiency.

Figure 1 depicts how the input, output, and contextual-business related variable sets are interrelated to each other, within the ambit of a learning or feedback process in the UK hotel industry, by means of information entropy and its derivate measures: joint entropy and mutual information. The underlying hypothesis is that super-efficiency scores are impacted by information entropy (for example., information dispersion of input, output, and contextual variables), joint entropy (for example., balanced joint variations of input, output, and contextual variable pairs), and mutual information (for example., information gains translated into operational synergy by means of managerial feedback or learning processes where it is possible to learn about one variable set through the behavior of the other).

The dataset summarized in Table 1 represents an unbalanced sample of 647 different hotels distributed among 153 distinct UK cities over the course of 10 years, from 2008–2017, totaling 1530 hotel-year (or Decision Making Unit-year) pairs of observations. Let’s consider *c* as the index for cities, *c* = {1 … *C*, *C* = 55}; *t* as the index for years = {1 … *T*, *T* = 10}; and *h* as the index for hotels = {1 … *H*, *H* = 153}. Let´s also consider that, for each hotel at each year of the sample, *i* = {1 … *I*; *I* = 3}is the index for its inputs (*x*); *o* = {1 … *O*; *O* = 5} is the index for its outputs (*y*); and *v* = {1 … *V*; *V* = 6} is the index for its contextual variables (*z*). At last, let *s* denote the (super) efficiency scores computed per each hotel *h* for each year *t*. Hence, the following information quantities can be defined:

Information entropy mass for the efficiency scores per hotel per year:

For efficiency scores:

(4) $$Em\left(S\right)=-s_{h,t,c}\log\left(s_{h,t}\right)\;\forall \;h,\;t,c$$

For super-efficiency scores:

(5) $$Em\left(S\right)=-s_{h,t,c}^{sup}\log\left(s_{h,t}^{sup}\right)\;\forall \;h,\;t,c$$

Information entropy mass at the hotel level per year per city:

For the inputs:

(6) $$Em\left(X\right)=-\frac{\sum_{i}x_{i,h,c,t}\log\left(x_{i,h,c,t}\right)}{I}$$

For the outputs:

(7) $$Em\left(Y\right)=-\frac{\sum_{o}y_{o,h,c,t}\log\left(y_{o,h,c,t}\right)}{O}\forall \;h,\;t,\;c$$

For the contextual variables:

(8) $$Em\left(Z\right)=-\frac{\sum_{v}z_{v,h,c,t}\log\left(z_{v,h,c,t}\right)}{V}\forall \;h,\;t,\;c$$

Conditional information entropy means per year per city:

For the inputs:

(9) $$E.\left(X\right)=-\frac{\sum_{i}\sum_{h}x_{i,h,t,c}\log\left(x_{i,h,t,c}\right)}{IH}\;\forall \;t,\;c$$

For the outputs:

(10) $$E.\left(Y\right)=-\frac{\sum_{o}\sum_{h}y_{o,h,t,c}\log\left(y_{o,h,t,c}\right)}{OH}\;\forall \;t,\;c$$

For the contextual variables:

(11) $$E.\left(Z\right)=-\frac{\sum_{v}\sum_{h}z_{v,h,t,c}\log\left(z_{v,h,t,c}\right)}{VH}\;\forall \;t,\;c$$

Conditional joint entropy per city per year:

For the input-output pair:

(12) $$JE.\left(X,Y\right)=\frac{\sum_{h}\left[\sum_{i}\sum_{o}x_{i,,h,t,c}y_{o,h,t,c}\log(\sum_{i}\sum_{o}x_{i,,h,t,c}y_{o,h,t,c})\right]}{OIH^{2}}\forall \;t,\;c$$

For the input-contextual pair:

(13) $$JE.\left(X,Z\right)=\frac{\sum_{h}\left[\sum_{i}\sum_{v}x_{i,,h,t,c}z_{v,h,t,c}\log(\sum_{i}\sum_{v}x_{i,,h,t,c}z_{v,h,t,c})\right]}{VIH^{2}}\forall \;t,\;c$$

For the output-contextual pair:

(14) $$JE.\left(Y,Z\right)=\frac{\sum_{h}\left[\sum_{o}\sum_{v}y_{o,h,t,c}z_{v,h,t,c}\log(\sum_{o}\sum_{v}y_{o,,h,t,c}z_{v,h,t,c})\right]}{VOH^{2}}\forall \;t,\;c$$

Conditional mutual information per city per year:

For the input-output pair:

(15) MI.(X,Y)=E.(X)+E.(Y)−JE.(X,Y)

For the input-contextual pair:

(16) MI.(X,Z)=E.(X)+E.(Z)−JE.(X,Z)

For the output-contextual pair:

(17) MI.(Y,Y)=E.(Y)+E.(Z)−JE.(Y,Z)

### 3.4. Least Absolute Shrinkage and Selection Operator Regression

The Least Absolute Shrinkage and Selection Operator relies upon the linear model but uses an alternative fitting procedure for estimating the coefficients *β*0, *β*1, …, *βp*. This procedure is more restrictive in estimating the coefficients, and sets a number of them to exactly zero when the tuning parameter *λ* (penalty) is sufficiently large [77]. Hence, in this sense the Least Absolute Shrinkage and Selection Operator is more interpretable than linear models, because in the final model the response variable will only be related to a small subset of the predictors. Selecting a good value of *λ* for the Least Absolute Shrinkage and Selection Operator is critical; and cross-validation technique is often used. Equation (18) gives the Least Absolute Shrinkage and Selection Operator model used in this research to select a subset of information entropy related variables capable of explaining UK hotel super-efficiency scores.

(18) $$\begin{array}{ll} \underset{t}{\sum }(\underset{h}{\sum }Em\left(s\right) & -\beta_{0}-\beta_{1}E.\left(X\right)-\beta_{2}E.\left(Y\right)-\beta_{3}E.\left(Z\right)\\ & -\beta_{4}Joint\;Entropy.\left(X,Y\right)-\beta_{5}Joint\;Entropy.\left(X,Z\right)\\ & -\beta_{6}Joint\;Entropy.\left(Y,Z\right)-\beta_{7}Mutual\;Information.\left(X,Y\right)\\ & -\beta_{8}Mutual\;Infomation.\left(X,Z\right)\\ & -\beta_{9}Mutual\;Information.\left(Y,Z\right))\\ & +\lambda \overset{9}{\underset{j=1}{\sum }}\left|\beta_{j}\right|=\mathrm{Residual}\;\mathrm{Sum}\;\mathrm{of}\;\mathrm{Squares}+\lambda \overset{9}{\underset{j=1}{\sum }}\left|\beta_{j}\right| \end{array}$$

## 4. Analysis and Discussion of Results

### 4.1. Results

**Figure 2:**
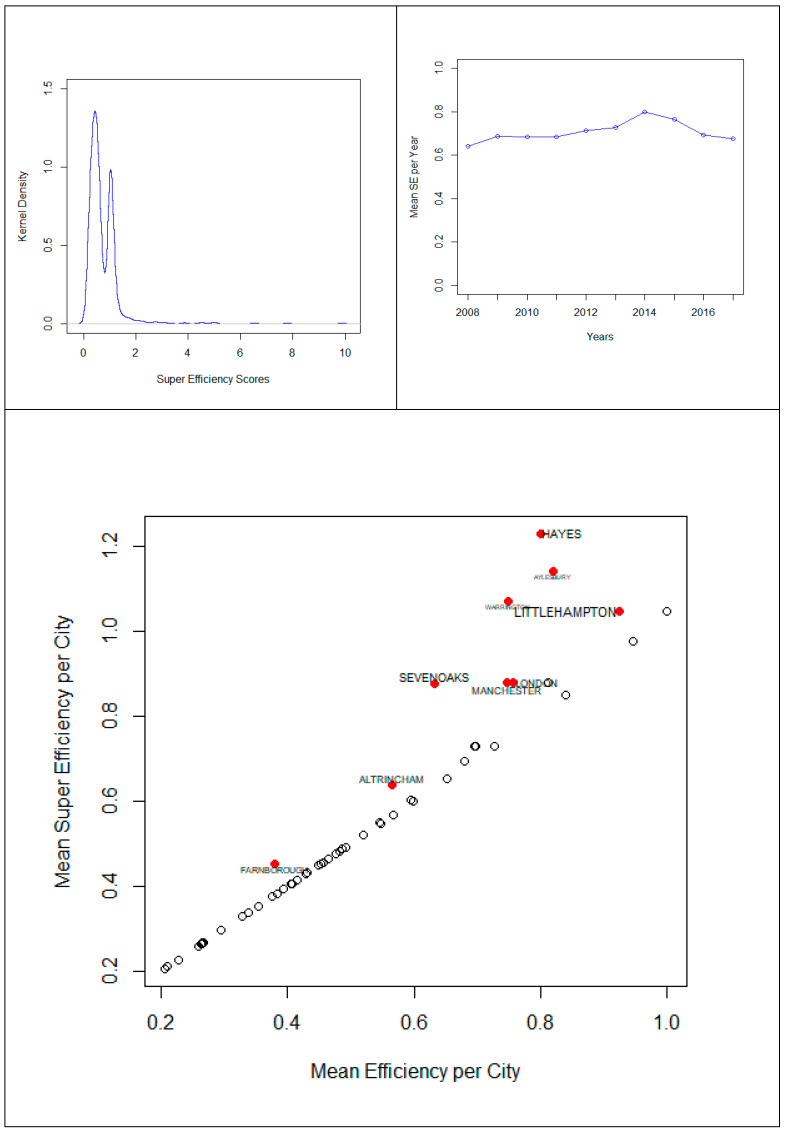
Super-efficiency scores distributions.

Results for the super-efficiency scores computed using the novel Modified Slack-Based Model are depicted in Figure 2 (upper left). The bi-modal shape of super-efficiency scores suggests the impact of distinct factors on UK hotel performance. As a matter of fact, super-efficiency scores appear not only to be not only withdrawing to lower levels after years of continuous increase (Figure 2 upper right), but also super-efficiency in UK hotels appears to be a phenomenon restricted to a small subsample of cities, including those big ones such as London and Manchester (Figure 2 lower). The reported efficiency level of the current study is much higher than the one reported by [21]. This is mainly attributed to the fact that these two studies used different time period and include different hotels in the sample and most importantly, [21] used the traditional data envelopment analysis, while the novel method we proposed benefits from the advantages of being able to address the issue of negative output in the analysis, which is supposed to provide more accurate and robust results. 

**Figure 3:**
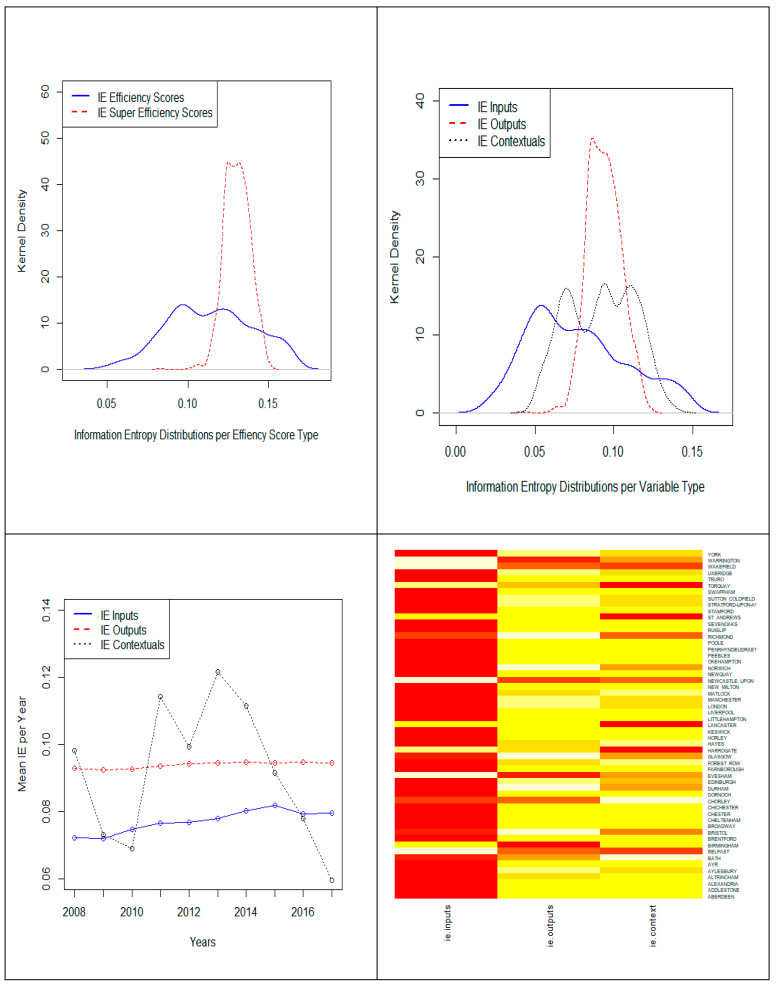
Information entropy distributions given different criteria.

The super efficiency observed for the hotels in the UK big cities can be attributed to the fact that big cities normally attract more inward tourist as well as outward tourist, in addition to this, normally large hotel chains will choose the big cities to operate their business, they can achieve the economics of scale. Besides, it is worth noting that this spatial-temporal heterogeneity captured by super-efficiency scores contributed to reduce the epistemic uncertainty on how hotel efficiency is geographically distributed in UK over the course of time. While mean information entropy slightly increased non-significantly at *p* < 0.05 from traditional Data Envelopment Analysis models to the proposed Modified Slack-Based Model (from 0.114 to 0.130), the dispersion of information entropy distributions was dramatically reduced (Figure 3 upper left) as long as efficiency scores are no longer asymmetrically concentrated towards 1.

It is also interesting to note that information entropy on inputs, outputs, and contextual variables also behave substantially different at the hotel level (Figure 3 upper right), a result that persists when analyzed by year (Figure 3 lower left) and city (Figure 3 lower right). 

Contextual, business-related, variables stand in between inputs and outputs in terms of information entropy dispersion, thus suggesting that, although not so balanced or quasi-uniformly distributed as the inputs at the hotel level, it is possible to learn from the market dynamics relative to touristic in and outflows as well as from the financial strength of hotel shareholders in UK, what may justify differences in cash flow generation potential and liquidity indicators.

**Figure 4:**
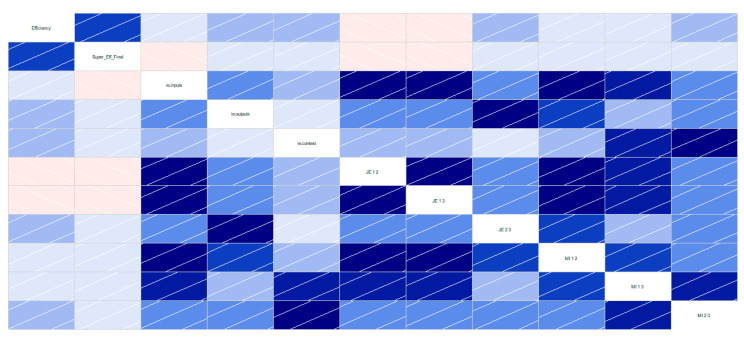
Information entropy, joint entropy, and mutual information correlogram.

Spatial-temporal analysis of information entropy reveals that, while output information entropy remained stagnant over the course of time, inputs and contextual variables somewhat evolved in distinct directions. As long as input information entropy increased, thus denoting that resource dimensioning has become a quite standardized planning at the hotel level, contextual, business-related, variables information entropy sharply decreased, therefore implying that market conditions with regard to tourism activity is increasingly offering, over the course of time, the opportunities for learning and improving hotel efficiency (Figure 3 bottom left and bottom right). As expected, a correlogram (Figure 4) among information entropy related variables indicates that information entropy, joint-entropy, and mutual information, although denoting different aspects of information reliability are strongly associated, what calls for further analysis in terms of informational efficiency drivers screening and selection. 

**Figure 5:**
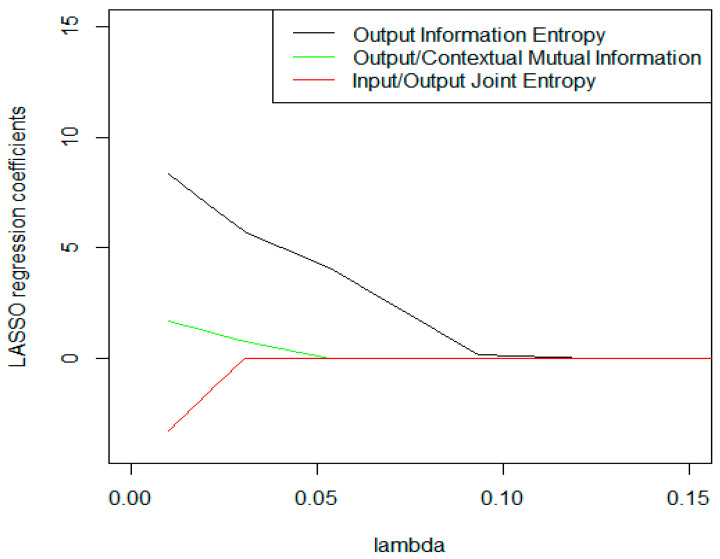
Least Absolute Shrinkage and Selection Operator regression results.

Least Absolute Shrinkage and Selection Operator regression results (Figure 5), following the cross-validation steps presented in [77], identified one information variable of each type as super-efficiency drivers in UK hotel operations: (i) output information entropy; (ii) output/contextual mutual information; and (iii) input/output joint entropy.

### 4.2. Discussions

As regards UK hotel productive resources, information entropy on inputs is much wider than that with regard to outputs. This means that not only proportionally less can be learnt from inputs than from outputs in order to improve efficiency levels, but also that resource allocation—number of employees, depreciation/amortization, and cost of goods sold—is more balanced—or quasi-uniformly distributed—at the hotel level than the respective cash flow and liquidity generated as a product of the operating size. This finding is new compared to previous empirical literature in hotel efficiency from the perspective we do not only identify the level of efficiency, but more importantly, we can derive the source of inefficiency from inputs and outputs. This also indicates that it would be more difficult for the UK hotels to optimize the resources in a better way, rather than thinking about the way of cutting the number of employees or reduce the cost of goods sold, UK hotels should focus more on how to increase the revenue, increase the level of liquidity and capitalization as well as reduce the volumes of expenses, which will be more effective in increasing the level of efficiency in the UK hotel industry.

We can also understand the result from the perspective that controlling the outputs is the most effective way to improve efficiency, while the general macroeconomic environment or the development of the international tourism industry will also have a significant impact on the efficiency level of UK hotels. This is in line with the findings of [78]. In addition, the financial structure in the UK hotel will also affect the hotel efficiency in the UK to a certain extent. However, [78] report that there was no significant impact of hotel financial structure (i.e., capitalization). This is mainly attributed to the fact that different methodologies were used in the analysis. more specifically, our propose of a Least Absolute Shrinkage and Selection Operator is more interpretable and also the regression benefits from the ability to select a subset of information entropy related variables capable of explaining UK hotel super-efficiency scores. 

Both variables (output information entropy and output/contextual mutual information) presented a positive impact on super-efficiency scores, indicating that the distinctive characteristic of superior performance in UK hotel operations is the capability to match the cash flow generation potential with market opportunities related to touristic in/outflow within the ambit of feedback learning processes. On the other hand, the negative result for input/output joint entropy reveals that input resource dimensioning imposes scale and revenue elasticity constraints in light of output optimization. 

As long as hotel operations are capital intensive, there is a great likelihood to exploit market opportunities, derived from changes in touristic flows by operating in decreasing returns to scale with continuous price discount policies to sustain liquidity and cash-flow generation of the course of time. This is exactly the case in the UK hotel industry, for instance, the hotels in the big cities such as London and Manchester, in the city center area, there would be a number of different hotels located near each other offering similar level/quality of accommodations/catering to the tourist, therefore we can deduce and imagine that the capacity of hotels to accommodate the tourists is big enough, increase the operational scale will not achieve the effect of cost saving, because of higher level of competition among different hotels, they should consider the measurements or owned policies to sustain their market share, one of the effect way would be reduce the price level through providing discount the customers which is supposed to attract more people. 

## 5. Conclusions 

The economic growth across the globe, together with the increase in the volume of Gross Domestic Product per capita make people have more disposable income. Besides the traditional physical products demanded and consumed by the people, there is a general trend of increasing in the consumption in the area of entertainment. In particular, people spent more time and money on exploring new experience (for example, experience new culture, new people, new food, new architecture, et cetera) through travelling to different countries, this is mainly derived from the globalization in which countries they do not only engage in trading with each other in tangible goods, but also in the areas of intangible services such as the international tourism. The United Kingdom, together with Germany, Italy and France, make up the four biggest economies in Europe. London, the capital city of the United Kingdom, is the world largest financial center, which does not only attract the influential financial institutions and companies to headquartered in, but also attract many people to look around and pay a visit to this legendary city and country every year. 

The hotel industry, one of the most important components of the hospitality sector facilitate the development of the country’s international tourism industry by providing accommodations and catering to both the local domestic people as well as the travelers from all over the world. The performance of the hotel industry is very important from the perspective that it will provide an indicator to the potential investors for their decision to invest the money in this sector. For instance, large amount of profit in this sector will attract more potential investors to enter into this industry, on the contrary, lower amount of profits will divert more people to invest the money to other sectors of the economy. For the existing hotel management and hotel practitioners, not only do they consider the profit through their operation, but also, they concern the issue of how to reduce the cost of operation through optimizing the resource allocation in the production process. In addition to this, both the hotel companies and academic researchers have a great interest in the factors which will influence the efficiency in the hotel industry, the latter devote a large amount of effort by proposing different types of advanced methods to investigate the efficiency of hotel companies. 

Different from the empirical studies in related to the estimation of efficiency in the hotel industry, we use a sample of UK hotels to significantly contribute to the area of efficiency analysis by proposing a modified slack-based model to evaluate the level of super-efficiency in addressing the issue of negative values of cash flow and liquidity variables. We are also the pioneer to propose the Least Absolute Shrinkage and Selection Operator to examine the influence of entropy related variables including both mutual information and joint entropy on efficiency scores. 

The findings of our study suggest that less can be learnt from the input side to improve the efficiency level in the UK hotel industry, while in comparison the results show that the market dynamics in related to tourism inflow and outflow together with financial strength of hotels in terms of total assets and shareholders’ funds have a higher explanatory power in the efficiency level in the UK hotel industry. We provide important policy implications to the UK hotel industry to improve the efficiency level: (1) the UK hotels should operate in a decreasing return to scale (reduce the size of operations) and also implement the policy of providing discount; (2) more focus should be given in the process of hotel management in terms of how to further increase the level of revenue, therefore, probably a diversification strategy could facilitate the achievement of this; (3) the UK hotels should further increase the liquidity level and also enhance the level of capitalization; (4) the UK hotels should pay attention to reduce the level of overhead costs, some of the areas that the hotels can focus on related to the cost reduction include the administration cost, utilities cost, insurance cost, marketing cost and maintenance cost. 

The future research could use alternative advanced Data Envelopment Analysis method to evaluate the efficiency in the UK hotel industry or use the advance Stochastic Frontier Analysis to check and compare the robustness of our results, while the robustness can be further checked by using alternative econometric techniques to test the influence of the contextual and business-related variables on the efficiency score. Finally, the sample could be expanded to including other European countries or compare the efficiency in the hotel industry between UK and other Asian countries. 

## Figures and Tables

**Table 1:** Basic descriptive statistics for UK hotels dataset.

**Variable Name**	**Number of Employees**	**Costs of Goods Sold**	**Depreciation Amortization**	**Operating Revenue**	**Other Operating Expenses**	**Liquidity Ratio**	**Capitalization**	**Size**
Variable type	x_1_	x_2_	x_3_	y_1_	y_2_	y_3_	y_4_	y_5_
definition	Labor	Cost of material and labor	Expenses of a fixed assets over its uneful life	Revenue generated by a hotel from accomodation services	Overhead expenses	Ratio between liquid assets and total assets	Ratio of shareholders’ funds and total assets	Natural logarithm of total assets
Mean	407.34	12,506.34	2246.88	32,224.33	16,233.96	1.83	0.30	4.69
Maximum	4124.00	167,886.12	23,236.49	364,911.94	213,270.02	33.54	0.98	6.31
Minimum	41.00	476.81	1.17	1382.78	(114,218.07)	0.00	(6.27)	3.00
Standard deviation	482.17	19,429.32	3180.72	41,993.19	23,784.11	3.15	0.60	0.52
coefficient of variation	1.18	1.55	1.42	1.30	1.47	1.73	2.05	0.11
**Variable Name**	**Number of Arrivals (international tourism)**	**Number of Departure (international tourism)**	**International Tourism (Expenditure)**	**International Tourism (Receipt)**	**Total assets**	**Shareholders’ Funds**		
Variable type	z_1_	z_2_	z_3_	z_4_	z_5_	z_6_		
definition	Overnight visitors who travel to the UK whose main purpose in visiting is not commercial.	number of departures that people make from the UK to any other country for any purpose other than a remunerated activity	Expenditure of UK tourists in other countries	Tourism receipt in the UK from tourists from other countries	All the items with economic value including cash, accounts receivable, inventory, equipment, tool, etc.	The amount of equity in a company, which belongs to the shareholders		
Mean	31,816,324.42	62,511,993.83	81.61	52.58	109,166.57	43,712.99		
Maximum	37,814,000.00	74,189,000.00	94.96	65.45	2,022,746.63	1,222,400.81		
Minimum	28,199,000.00	55,562,000.00	71.67	39.04	1008.69	(288,298.06)		
Standard deviation	3,566,900.57	6,458,211.21	7.40	8.69	204,176.45	123,073.49		
Coefficient of variation	0.11	0.10	0.09	0.17	1.87	2.82		

**Table 2:** Data of Decision Making Units.

Decision Making Unit	$x_{1}$	$x_{2}$	$y_{1}$	$y_{2}$	$y_{3}$
A	1.03	−0.05	0.56	−0.09	−0.44
B	1.75	−0.17	0.74	−0.24	−0.31
C	1.44	−0.56	1.37	−0.35	−0.21
D	10.8	−0.22	5.61	−0.98	−3.79
E	1.3	−0.07	0.49	−1.08	−0.34
F	1.98	−0.1	1.61	−0.44	−0.34
G	0.97	−0.17	0.82	−0.08	−0.43
H	9.82	−2.32	5.61	−1.42	−1.94
I	1.59	0	0.52	0	−0.37
J	5.96	−0.15	2.14	−0.52	−0.18
K	1.29	−0.11	0.57	0	−0.24
L	2.38	−0.25	0.57	−0.67	−0.43
M	10.3	−0.16	9.56	−0.58	0

**Table 3:** Efficiency, Super Efficiency and rank for Example 1.

Decision Making Unit	Efficiency	Super Efficiency	Rank
A	0.84134		6
B	0.71002		10
C	1	1.11675	5
D	0.63821		12
E	0.72132		9
F	0.77915		8
G	1	1.2436	3
H	1	7.55694	1
I	0.80078		7
J	0.69683		11
K	1	1.21105	4
L	0.61005		13
M	1	2.9725	2
